# Lessons learned in the development of process quality indicators for cancer care in Japan

**DOI:** 10.1186/1751-0759-4-14

**Published:** 2010-11-05

**Authors:** Takahiro Higashi

**Affiliations:** 1 Department of Health Policy/Public Health, Graduate School of Medicine, the University of Tokyo, Tokyo, Japan

## Abstract

In Japan, attention has increasingly focused on ensuring the quality of care, particularly in the area of cancer care. The 2006 Basic Cancer Control Act reinforced efforts to ensure the quality of cancer care in a number of sectors, including the role of government in ensuring quality. We initiated a government-funded research project to develop quality indicators to measure the quality of care for five major cancers (breast, lung, stomach, colorectal, and liver cancer) in Japan, and palliative care for cancers in general. While we successfully developed a total of 206 quality indicators, a number of issues have been raised regarding the concepts and methodologies used to measure quality. Examples include the choice between measuring the process of care versus the outcome of care; the degree to which the process-outcome link should be confirmed in real-world measurement; handling of exceptional cases; interpretation of measurement results between quality of care versus quality of documentation; creation of summary scores; and the optimal number of quality indicators for measurement considering the trade-off between the measurement validity versus resource limitations. These and other issues must be carefully considered when attempting to measure quality of care, and although many appear to have no correct answer, continuation of the project requires that a decision nevertheless be made. Future activities in this project, which is still ongoing, should focus on the further exploration of these problems.

## Introduction

In Japan, interest in ensuring the quality of patient health care has recently increased. Although Japanese citizens have enjoyed universal health insurance coverage for more than 40 years, concern has been expressed at the lack of an efficient system for monitoring the quality of care[1,2]. To date, quality monitoring has been sporadic at best[3,4], and organized efforts to improve quality have yet to be established. In the area of cancer care, this concern led to the enactment of the Cancer Control Act in 2006, which mandated that the government adopt a leadership role and take responsibility in ensuring the quality of cancer care nationwide[5]. However, ensuring quality care first requires an efficient means of measuring it.

To this end, in 2006 the Japanese government funded a research project aimed at developing a system for measuring the quality of cancer care, focusing primarily on the five major types of cancer in Japan, namely breast, lung, stomach, colorectal, and liver cancer, as well as palliative care. Given that the project was aimed at measuring quality in terms of how current best practice was applied, rather than the general suitability of services (such as waiting times or the comfort of hospital beds), we sought extensive involvement from nationally recognized clinical experts in respective clinical areas. Methodology was under the direction of an epidemiologist and a health services researcher, while the contents of quality measurement were primarily defined by clinicians.

An overview of the study was published in Japanese[6]. Briefly, we developed a total of 206 process-of-care quality indicators for cancer care using methodology developed by the researchers at University of California, Los Angeles and RAND Corporation[7-12]. This involved creating a set of candidate quality indicators, compiling evidence to support these indicators, and having a panel of multidisciplinary experts examine them for validity in two rounds of rating (scale of 1-9), once before and once after a face-to-face discussion. After discarding those indicators judged as having low validity, 206 indicators remained, all of which described the standards of care which define the target patients and the care processes that need to be provided to them. Example indicators are presented in Table 1.

**Table 1:** Example of quality indicators developed in our project

Denominator (target patients)	Numerator (care processes indicated)
Patients with stage 3 colorectal cancer who have undergone surgical resection	Patients who have received standard chemotherapy within 8 weeks of surgery or who have reasons for not receiving chemotherapy
Patients with colorectal cancer who have undergone surgical resection	Patients who have received total colonoscopy or who have reasons for not receiving total colonoscopy

Patients with gastric cancer who have undergone endoscopic resection and have one of the following: • positive vertical margin • lymphovascular invasion • invasion to the SM2 layer (≥500 μm)	Patients who have received surgical treatment with lymph-node dissection
Patients diagnosed with breast cancer	Patients who have had their Her-2/neu status examined
Patients receiving treatment for liver cancer	Patients who have had the levels of α-fetoprotein and protein in vitamin K absence-II checked before the start of therapy
Patient with lung cancer who have undergone surgical resection or radiation therapy on their lungs	Patients whose lung function has been assessed via spirometry
Patient receiving narcotic analgesics	Patients who have received education or medication to prevent constipation

Since most Japanese clinicians have no experience with quality measurement, such assessment must be approached with care, while conscientiously addressing a number of major issues. Although the study is still underway, we describe here several issues of note and our methods of addressing them in the hope that they may be of value to researchers and policy makers developing similar quality measurement systems. Before continuing, however, we caution that there are no right solutions or answers to these issues and questions and instead remind readers that the decisions made here were those which we believe best served the present purpose.

## Choosing to examine process vs. outcome

Quality of care is typically measured with regard to structure, process, or outcome[13,14]. Structural quality refers to organizational and facility quality, such as the number of specialists on staff, staff-patient ratio, and the availability of high-technology equipment. Process quality refers to the appropriateness of care provided during patient encounters, such as the appropriateness of medications and the selection of therapies and follow-up. Outcome measurement estimates the quality of care that patients receive by examining what happens to patients as a result of care, such as mortality after surgery and readmission after discharge.

Establishing a quality monitoring system first requires a decision on which level(s) should be addressed, taking into consideration the relative strengths and weaknesses of each. Given that the government has already enacted structural requirements for hospitals seeking designation as "designated cancer care hospitals,"[15] the choice for the present quality measurement lies between process and outcome. Outcome in cancer care is typically measured by five-year survival[16,17], and given that improvement of outcome is the general objective of medicine, its importance is clear. Member hospitals of the Japanese Association of Clinical Cancer Centers *(Zen-gan-kyo) *acknowledged this in 2007 through their initiation of public reporting of five-year survival rates by facility[18].

However, outcome reflects not only the medical care provided but other factors as well, such as patient baseline health and compliance with medical advice. Any comparison of quality across populations and facilities or over time needs to statistically adjust for these factors, particularly patient-case mix[19]. Moreover, the calculation of five-year survival is necessarily a five-year process, a time lag which weakens the measurement's value in improving care[13].

In contrast, evaluation of process, involving the specific focus on what is done to patients, is the most direct of the three measurement schemes. Process quality indicators describe what should be done to what type of patient under which clinical conditions, and the results accordingly highlight direct targets for improvement. Unlike outcome, process does not require a lengthy five-year period to obtain results once the measurement system is established. Indeed, when integrated with electronic order entry and medical records, process values can even be used prospectively, as clinical reminders[20].

One challenge to using process measures is criteria definition. Although quality indicators should be based on clinical evidence, direct clinical evidence among important populations (e.g. older persons) is occasionally limited[10], and criteria must therefore be extrapolated from studies in other populations (e.g. younger persons) based on expert consensus. In this situation, we considered that the optimal methodology to examine the consensus was that developed by the RAND Corporation, as described above[7-12]. While this methodology is not perfect[21], we expect that it will provide the least biased set of quality indicators when appropriately performed, given previous findings that quality criteria developed using this methodology is reproducible[22,23] and agree with the opinions of practicing physicians[24] and have predictive validity[25].

## Proving the process-outcome link

To qualify as a valid process quality indicator, a particular standard of care should help to improve patient outcome; if it does not, the provision of such care cannot be considered high-quality. Donabedian called this the "contributional validity" of the process measure[13]. The lack of direct clinical evidence from randomized controlled trials encountered in many clinical situations sometimes renders contributional validity ambiguous, and clinicians can present different interpretations of evidence from those of the expert panel members who helped to develop an indicator, particularly with indicators developed based on indirect evidence. Typical examples of such lack of direct evidence occur with quality indicators that target diagnostic standards: although computer tomography (CT) of the liver before colon cancer surgery is a standard of care, for example, on the basis that CT scan results affect treatment decisions and can thus be a quality indicator, no randomized controlled trial has examined the utility of CT of the liver before surgery. Indeed, few diagnostic procedures have been subject to randomized controlled trials.

Several points must be carefully considered before examining the relationship between the process quality measured and outcomes. First, the unit of analysis must be identified. When comparing outcomes of patients who do or do not receive a certain therapy, for example, the patient is the unit of analysis[25,26]. In comparing adherence rates, in contrast, the facility is the unit[27,28]. Although the relationship at the aggregate level does not necessarily imply the relationship at the individual level, owing to the *ecological fallacy*[29], analysis at the facility level will have acceptable validity if it is accepted that the adherence rate represents the quality of care provided by the facility, and incorporates other aspects of care than those specifically measured in the quality indicators. The appropriate unit of analysis will depend on the unit's comparability and the level at which the quality data are available.

Second, as quality indicators may target different types of outcomes, the outcomes themselves must be carefully chosen[14]. The most common patient outcome in cancer care is five-year survival, but not all high-level care prolongs life, or aims to prolong life. To list three examples, suitable explanation of the risks and benefits of treatment options is surely an aspect of quality, specifically the provision of respect to patient autonomy, but it is unlikely to improve survival; administration of anti-emetic medications before chemotherapy is aimed at alleviating adverse symptoms during therapy, and not to make any contribution to survival; and patient education about drug regimens may increase compliance or reduce medication errors. Although these may improve patient health or prevent adverse events, their benefits may be too small to detect from observation, yet they are strongly supported as quality indicators.

Another point in considering the process-outcome link may be the timing of outcome observation. This is seen in any epidemiological study which examines the cause-effect relationship[29]: outcomes of acute conditions may be observed relatively early, while preventive care processes will require some time to show results. Evaluation of 100-year survival will show no impact of quality no matter how high it may be. Though absurd, this example highlights the importance of timing, and the difficulty often met in determining the right timing for a particular outcome.

Finally, interpretation of process-outcome links observed in the real world requires caution. As randomization of patients to high or low quality care is ethically untenable, studies examining quality-outcome relationships are necessarily observational; and since persons receiving high- and low-quality care may differ, the level of evidence from such studies is lower than that from randomized controlled trials, where optimum comparability can be expected. If the content of a quality indicator (i.e., standard of care) is supported by randomized controlled trials, its validity as an indicator may not be refuted by a lack of relationship to outcomes observed in clinical application, unless the target population is extremely different.

## Handling exceptions

No rule is free of exceptions. Although quality criteria define which patients should receive which care processes, some patients will not receive a particular item of care for any of a number of reasons: for example, patients with acute myocardial infarction should, by standard, receive aspirin on admission, but this is of course foregone if the patient is allergic to aspirin. In this case, "high quality" can be defined as care that appropriately distinguishes exceptions from regular cases and tailors treatment to the individual patient. Unfairly penalizing such cases as "failures" to provide standard care should be avoided.

Three methods of handling exceptions to quality indicators can be considered. First, these cases can be excluded from the denominator in calculating the adherence rate, thereby providing a "pure" sample of standard patients. In our study also, denominators for some indicators were made narrower than the population to which the numerator care is usually applied in regular practice. This is because we wanted a sample to whom the care is clearly applicable. However, this method not only fails to give proper credit to the provider who makes these important decisions of tailored medicine, but also lowers the observed quality score (i.e. adherence rate to the quality indicator), because subtracting the same number from both the numerator and denominator actually decreases the fraction when the fraction is less than 1 (for example, (8-1)/(10-1) = 7/9 = 0.78 is less than 8/10 = 0.8). Further, relatively small target populations can render quality scores statistically unstable, with large standard errors.

A second method of handling exceptions is to treat them as if the care had in fact been provided, provided that the reason for the exception is properly documented. In this way, atypical patients can be kept in the sample and the care they receive can be evaluated in the same way as for regular patients. Quality scores using this method tend to be more stable than those using the first method above, but patient eligibility for the quality indicator becomes heterogeneous, which may then dilute the link between the quality and the outcome by introducing noise into the biological process-outcome link. While acknowledging this limitation, we basically adopted this second method of handling exceptions in the present study. This can be seen in the fact that many of our quality indicators explicitly demand documentation of the reason why the indicated care was not provided (Table 1).

The third way of handling exceptions is to reduce target adherence by the expected number of exception patients[30]. If 5% of patients ostensibly applicable to a quality indicator are estimated to become exceptions, the quality target can be set at 95% instead of 100%. Given that identifying individual exceptions requires that the researcher abstracting the medical record has knowledge of treatment choices, which is occasionally difficult due to a lack of detailed documentation[31], a simple reduction in target enables the judgment of individual cases to be avoided, and also thus the risk of errors in such judgment. Further, gaming with the system can be discouraged by labeling patients who did not receive the indicated care as exceptions, perhaps retrospectively. In comparison of quality scores across populations, however, national variations in the proportion of exception cases may introduce large random errors into the comparison.

The above clearly demonstrates the difficulty in handling exceptions. Even when considering only individual cases, we may well refrain from stating that the target quality score is 100%, or instead state outright that the target is slightly lower than 100%. Indeed, the UK pay-for-performance initiative (Quality and Outcomes Framework) gives full points even for quality scores less than 100%[32,33], although they also allowed providers to limit the denominators reporting exceptions. At the least, quality information should be interpreted together with data regarding the nature and number of exceptions reported by providers[30,32,33].

## Quality of care vs. quality of documentation

Quality scores depend to some extent on the quality of available data. While process-of-care quality should ideally be evaluated via direct observation, or perhaps in standardized patients[34], information is typically collected from medical records[9,35-37]. In addition, documentation of essential clinical information, such as cancer stage and follow-up review of drug regimens, consists of quality indicators based on the notion that such documentation represents an aspect of quality of care[31,38]. Both a reliance on documentation for the implementation of quality indicators and a specific focus on documentation among several quality indicators leads to the impression that quality measurement overly emphasizes documentation, and thereby begs the question of whether quality evaluation measures the quality of medical care or the quality of documentation.

Physicians who believe that documentation is a largely separate issue from medical care may be reluctant to accept that pertinent documentation is part of quality care. Indeed, during our informal discussion about quality indicators, some clinicians practicing in urban areas gave the example of providers in rural areas where a small number of health professionals handle most medical issues. They noted that rural physicians may feel particularly strongly about this aspect, as they are usually too busy to keep detailed records and may not need to communicate with the few other health professionals in their area through documentation, opting instead for face-to-face conversations. However, having sufficient documentation ensures smooth sharing of information among health care professionals, supplementing face-to-face conversation. Given that a miscommunication is a major cause of medical errors[39], quality of documentation can be safely deemed as falling under the umbrella of quality of care, which will thereby increase the probability of safe practices and improved outcomes.

## Scope of quality measures and number of indicators

Medical care has various aspects, including not only medical intervention but also patient education and coordination of providers, and the long continuum spanning prevention, diagnosis, treatment, and follow-up. Narrow evaluation captures only limited aspects and cannot represent the quality of care[40]. In addition, evaluation based on a small number of quality criteria can easily be gamed by providers allocating resources only to satisfy the defined quality indicators and achieve high scores, leaving out or even sacrificing other, perhaps more important aspects of care[41].

In contrast, broad measurement of quality covering various aspects of care is resource- and labor-expensive. Insurance claims data can be a suitable alternative, provided the necessary information is available; the range of information is limited to the original utilization, however, and the data tend to lack details regarding patient condition, such as laboratory and imaging results[42]. An empirical study examining broad aspects of quality based on medical records found that quality indicators that can be measured from claims data tended to result in better scores than those requiring information from medical records[43]. This finding suggests that selecting quality indicators based on the availability of information in insurance claims may fail to detect and solve problems in quality of care. Instead, theoretically, selection should be primarily based on the importance of the care process. Significant attention must be paid to the balance between the validity of the quality measurement and the resources spent on measurement.

## Quality indicators in rare but important situations

Several issues must be considered when selecting quality indicators by the priority of measurement. One such issue is the expected effectiveness of the care indicated in the quality measure, i.e. the potential for the care to improve the patient's outcome. Another issue is the expected room for improvement in the practice being evaluated; care processes which are known to be always performed, for example, do not need to be re-examined.

A particularly controversial perspective is the number of patients in whom the quality indicator is applicable. One example is the need to perform additional surgical resection after endoscopic resection of a cancer which turned out to be more deeply extended than initially estimated, with a high risk of lymph node metastasis. Given that such cases are relatively rare, however, including these quality indicators in the measurement set may be inefficient, because information needs to be collected from the entire target patient population to determine whether the quality indicator is applicable to them. In addition, when the number of applicable patients is small, the denominator of the quality score as the percent adherence is also small, making scores calculated using adherence unstable. This latter problem can be solved by obtaining a larger denominator through application of the indicator to a larger population or for a longer duration; for example, even if one quality indicator applies to only three patients in a hospital annually, it may be applicable to 30 patients in a town with 5 hospitals over a 2-year period. The importance of a quality indicator should therefore be judged on a global scale; if the care process described in the indicator strongly affects even a few patients, a decision should be made based on contextual factors, such as whether or not the indicator can be applied on a broader level to achieve a sufficient number of applicable patients.

## Creating a summary score

Quality indicators are scored based on the percentage of patients who receive the care described in them. Our cancer project produced 206 quality scores. When reviewing the results, the research team felt that interpreting such many scores was difficult. To summarize them, we therefore produced an overall number of adherence to the indicators by dividing the total number of patients to whom the quality indicator care was provided (sum of all numerators) by the total number of times the quality indicator was applicable to the sample patients (sum of all denominators). Although this score has the conceptually reasonable meaning of "overall performance of standards by the provider," we then speculated whether all indicators should have equal weight in calculating the summary score, given that quality indicators appeared to have different degrees of importance. In particular, for those who do not think documentation is important, documentation indicators must have smaller weights than other indicators.

Creating summary score weighting for quality indicators presents a challenge[44]. While the overall performance is one-way, this orientation ignores the natural importance of the care processes. An indicator's weight can relate to the comparative importance of the care process; for example, providing oxygen to a hypoxic patient is more important--at that moment--than documenting stage of cancer within one month of diagnosis.

Assessment of comparative importance can be aided by reviewing the expected improvement in outcome if the care is provided. However, when quality indicators target different stages of management of a disease, the comparison requires clinical judgment. For example, when determining whether or not prescribing anti-emetic medications before starting chemotherapy is more (or less) important than examining the entire colon before surgery, we must consider the difference in the basic nature of the outcomes expected to be improved by these care processes. Clinical judgment can be elicited either directly in the context of quality measurement by expert ratings of overall importance of quality indicators, as done by Ashton et al[45]., or by focusing solely on outcome by asking clinicians about the overall degree of outcome improvement on a single scale, integrating the expected different outcomes such as survival and quality of life, as is done by the indicator selection at the National Comprehensive Cancer Network[46], albeit that this is not to assign weights but to rank quality indicators by their priority. Future studies should assess the validity and reliability of such methodologies.

One way to circumvent the difficulty in assigning weights is to create a rule which integrates multiple quality indicators, such as the all-or-none rule[47]. Under this rule, several quality indicators are grouped together, and patients who receive all the indicated care according to the quality indicator set are counted. For example, if a patient is eligible for quality indicators A, B, and C, s/he is counted as having received quality care only if s/he has received all the care described in indicators A, B, and C. If s/he receives care in quality indicator A and B, but not C, s/he is not counted as having received the care. The quality score for a facility or patient group is then defined as the proportion of patients who received "perfect" care in the quality indicator set. Use of this strategy is supported by the notion that care is interrelated, and that only receiving all necessary care in the target area is acceptable. However, the score then depends on the grouping of the quality indicators, which must be theoretically justifiable, and most importantly, determined before the start of measurement. Further, if multiple such groups are created, weighting of scores may again become an issue, presenting new problems. In addition, handling of exceptions to quality measurement becomes increasingly important, as mistakenly entering one quality indicator event that should have been excluded reduces the whole count for a patient.

In our present study, we have not yet decided on the best method of calculating the summary score. Several approaches will likely need to be tested, and discussions among members of the research team and related clinicians will be conducted to address the possible options.

## Conclusion

A number of issues must be addressed when developing process quality indicators for cancer care, several of which are reviewed here. While our project specifically targets cancer care, many of these issues may also apply in other clinical areas. Future researchers should not expect to find the "right answers" to these issues and questions, but rather should make decisions based on best judgment, and thereby ensure progress. While different systems can call for different decisions, comparison of quality across patient populations (e.g., several facilities or over time) requires recognition of the fact that these decisions must be consistent across the compared groups.

## Competing interests

The author declares that they have no competing interests.

## Authors' contributions

The author wrote the manuscript and hold responsibility for the content of the manuscript.
